# Non-invasive prenatal testing – new possibilities on the horizon and old problems

**DOI:** 10.1515/medgen-2026-3028

**Published:** 2026-11-06

**Authors:** Anja Weise, Thomas Liehr

**Affiliations:** Jena University Hospital Institute of Human Genetics Am Klinikum 1 07747 Jena Germany; Jena University Hospital Institute of Human Genetics Am Klinikum 1 07747 Jena Germany

**Keywords:** non-invasive prenatal testing (NIPT), pregnant woman perspective, false-positive NIPT results, false-negative NIPT results, education programs

## Abstract

Non-invasive prenatal testing (NIPT) is a screening method that accesses placenta-derived DNA. Contrary to its name, no foetal DNA is tested here, despite the fact that it is often referred to as a test of ‘free foetal DNA’. NIPT is available in at least five different technical variants and can test for aneuploidies of chromosomes 13, 18 and 21, with or without testing the sex chromosomes. It can also test all chromosomes for aneuploidy, although polyploidy is only accessible with a single nucleotide polymorphism (SNP)-based test system. It can also check for targeted microdeletion/microduplication syndrome critical regions. Among others, as NIPT uses placental DNA, a certain rate of false-positive and false-negative results is to be expected in the case of copy number variation detection. For some years now, existing commercial NIPT variants have been expanded to include single-gene-mutation-oriented testing. Here, foeto-placental divergence seems to play a minor role, while distinguishing maternal and placenta-derived cell-free DNA remains challenging. This article reviews the milestones of NIPT and its current developments, focusing on the challenges of incorporating this approach into existing prenatal care schemes and the situation in Germany.

## Introduction

Prenatal diagnostics (PD) was introduced on a routine basis between the 1980s and 1990s. Although the first non-invasive test method of PD, ultrasonography, was developed in the late 1950s, invasive PD procedures such as chorionic villus sampling (CVS) from the placenta and amniocentesis (AC) were initially performed without visual control [1]. A third variant of invasive PD testing involves acquiring umbilical cord blood (UBC). The history of invasive and non-invasive PD, with a focus on current non-invasive prenatal testing (NIPT) developments, is summarised in Table 1 [2–12].

While invasive PD testing provides a genotype or karyotype of foetus, such as a karyotype of 46,U or 47,U+21, non-invasive PD tests only offer an estimation of risk [13]. Major foetal structural anomalies in sonography may also indicate a heritable disorder, but only a suspected genetic diagnosis can be inferred (see Table 1). The most prevalent sonographic marker is enhanced nuchal translucency (NT). The logic behind suspecting a genetic disorder when observing this in sonography is as follows: impaired normal function of the heart, kidneys or lymph system leads to the foetus having difficulty getting rid of tissue fluid, which accumulates under the skin – specifically in the neck region. Therefore, if enhanced NT is observed, it may indicate an underlying organic problem, as organ malfunction can but must not be caused by genetic factors. Therefore, NT is often considered an indication of a genetic disorder such as Down syndrome, Turner syndrome or Noonan syndrome [1].

Traditionally, non-invasive prenatal (PD) tests include sonography and biochemical testing of maternal blood, mainly performed during the second trimester (see Table 1). Note that the acquisition of maternal blood, which is an invasive procedure, is not considered an invasive PD testing method as it does not cause injury to the foetus or embryonic tissues [1]. The biochemical parameters tested in maternal blood are alpha-foetoprotein (AFP) total hCG, and unconjugated estriol in second trimester, and free beta-human chorionic gonadotropin (beta-hCG) and pregnancy-associated plasma protein A (PAPP-A) in first trimester, also known as the ‘triple test’. While these biochemical parameters can be statistically associated with pregnancy outcomes, they can also produce false positives and false negatives in individual pregnancies. As most data is available for children with Down’s syndrome, the risk estimation is usually given as the individual risk of this chromosomal disorder. Ultimately, an abnormal beta-hCG value may be present if the child has trisomy 13 or a microdeletion syndrome [1].

First trimester screening, which combines data such as maternal age and ethnicity with biochemical parameters from maternal blood and sonographic results to provide an overall risk estimation for the individual pregnancy, was introduced in the early 2000s (see Table 1). This test is not specific to one or several genetic disorders. If the results are abnormal, first trimester screening indicates the need for an invasive PD, which can identify aberrations such as whole or partial chromosome imbalances, microdeletion or microduplication syndromes [14], and monogenic disorders, e.g. Noonan syndrome [15], depending on the methods used.

**Table 1:** Timeline for important developments of prenatal diagnostics (PD) with focus on NIPT.

Performed first time [Ref]	Invasive	Type of test	Target fetus = fet maternal = mat placenta = pla
1956 [2]	yes	Amniocentesis (proof of principle)	fet
1950s to 1970s [3–5]	no	sonography (proof of principle)	fet
~1980 [4–5]	no	sonography (routine for malformations)	fet
~1980 [4; 6]	no	Biochemical tests from maternal blood, 2^nd^ trimester (routine)	mat / pla
1983 [7]	yes	Chorionic villus sampling (proof of principle)	pla
1983 [8]	yes	Umbilical cord blood (proof of principle)	fet
~1990 [4]	no	sonography (routine for prenatal care at 20 w. o. g.)	fet
early 1990s [4]	yes	Chorionic villus sampling (routine)	pla
early 1990s [4]	yes	Umbilical cord blood (routine)	fet
1997 [9]	no	NIPT Discovery of cff-DNA in maternal blood	pla
1998 [10]	no	NIPT Fetal rhesus D (RhD) status in RhD negative mothers (research)	pla
2000 [3]	no	NIPT for skeletal dysplasia (research)	pla
2002 [3]	no	NIPT for prenatal exclusion of cystic fibroses and other recessive disorders (research)	pla
early 2000s [4; 6]	no	First trimester screening (routine – based on biochemical tests from maternal blood, maternal age and sonography)	fet / mat
~2005 [10]	no	NIPT for RhD status (routine)	pla
2008 [3]	no	NIPT for single gene disorders (research)	pla
2011 [4; 11–12]	no	NIPT for fetal sexing (routine)	pla
2013 [4]	no	NIPT (+13, +18, +21, SCA) (routine)	pla
2017 [4]	no	NIPT (+13, +18, +21, SCA, RAT) (routine)	pla
2018 [3–4]	no	NIPT (+13, +18, +21, SCA, single gene mutations) (routine)	pla
2021 [3]	no	NIPT – discovery of cff-DNA fragments suited for long read sequencing (research)	pla

While non-invasive approaches offer the advantage of avoiding definitive diagnoses and only providing risk estimates, invasive procedures are associated with an abortion risk. Initially, mainly due to the larger diameter of the needles used to access the foetus in the early years of AC, CVS or UBC sampling, the abortion rates were ~1 % to 3 %. By 2010 at the latest, these risk figures had dropped to 0.1 – 0.3 % in Western countries [16]. It is also important to understand that the three basic invasive PD tests access different tissues: AC mainly accesses skin, bladder and colon-derived foetal cells, and UBC accesses foetal blood cells, whereas CVS accesses placental tissue. It is well known that foeto-placental discordance (confined placental mosaicism, or CPM) can occur in up to two percent of cases, as maternal cell contamination [13, 16].

NIPT has been used as a non-invasive screening tool for specific genetic disorders since 2011. Like other non-invasive screening tests, it relies on sampling maternal blood. Like CVS, NIPT does not access foetal cells, but rather placenta-derived trophoblasts. Therefore, it has the same principal restrictions as CVS: it may produce false positive or false negative results due to CPM and other reasons. Furthermore, the most widely used NIPT approaches only detect specific chromosomal imbalances (see Table 1). This means that a negative NIPT screening result for trisomies 13, 18 and 21, and sex chromosome aberrations (SCA), still misses ~50 % of pregnancies with other possible imbalances [13, 16]. To reduce this diagnostic gap, gynaecological guidelines recommend invasive PD in cases involving foetal sonographic markers [17].

Even though the placental origin is clearly mentioned in the first paper about NIPT, the authors of this paper decided to refer to the free, placenta-derived DNA as ‘cell-free foetal DNA’ (cffDNA) [9]. Unfortunately, this misleading term is still in use and hinders understanding of why false negative and false positive results are possible in NIPT. Cff-DNA is present in maternal plasma alongside cf-DNA, which is derived from apoptotic maternal cells. The amount of cff-DNA compared to maternal cf-DNA is referred to as the ‘foetal fraction’ [13]. The different lengths of cff-DNA (~142 bp) and cf-DNA (~166 bp) are under discussion as a means of distinguishing between the two types of DNA in NIPT [18], while some tests are based on single nucleotide polymorphisms (SNPs) to distinguish between maternal and foetal DNA fragments [19].

## Different NIPT approaches

When checking PubMed or Google for NIPT studies, a newcomer to the field or a layperson may get the impression that ‘NIPT’ is a clearly defined approach. It is easy to think that one NIPT study is the same as another, as most research or review articles do not mention technical or target-specific differences in the abstracts [13, 16].

However, NIPT approaches can differ in many ways. For example, they may be based on different techniques and may or may not be able to distinguish between paternal-, maternal- and placenta-derived DNA. They may also target different regions of the human genome (see Table 1).

### The technical basis of the differences between NIPT approaches and their consequences

In a recent review, Marton et al. [20] identified the four approaches currently used for aneuploidy screening in NIPT:

a) Whole-genome sequencing of cfDNA without enrichment
b) SNP-based PCR amplification of cfDNA followed by next-generation sequencing
c) Amplification and fluorescence labelling of cfDNA fragments for application to microarrays
d) Rolling circle amplification and fluorescence labelling of specific cfDNA fragments to detect aneuploidy by measuring fluorescence intensity subsequently.

According to their study, all of these approaches perform similarly in terms of clinical sensitivity, specificity and negative predictive value. However, methods b) and d) had lower positive predictive values than methods a) and c). A fifth approach — target capture sequencing NIPT — is mentioned by Kypri et al. [21], who also provide data on which target regions are covered by which NIPT approach. Hanson et al. [3] discuss variants of these five approaches in more detail.

Accordingly, consumers of NIPT must be made experts during genetic pre-test counselling about the type of test applied. This is particularly important because only SNP-based tests can detect triploidy [19], which has a prevalence of 0.03 % between weeks 10 and 14 of gestation, based on 1^st^ trimester ultrasonography and biochemical screening results [22].

**Figure 1:**
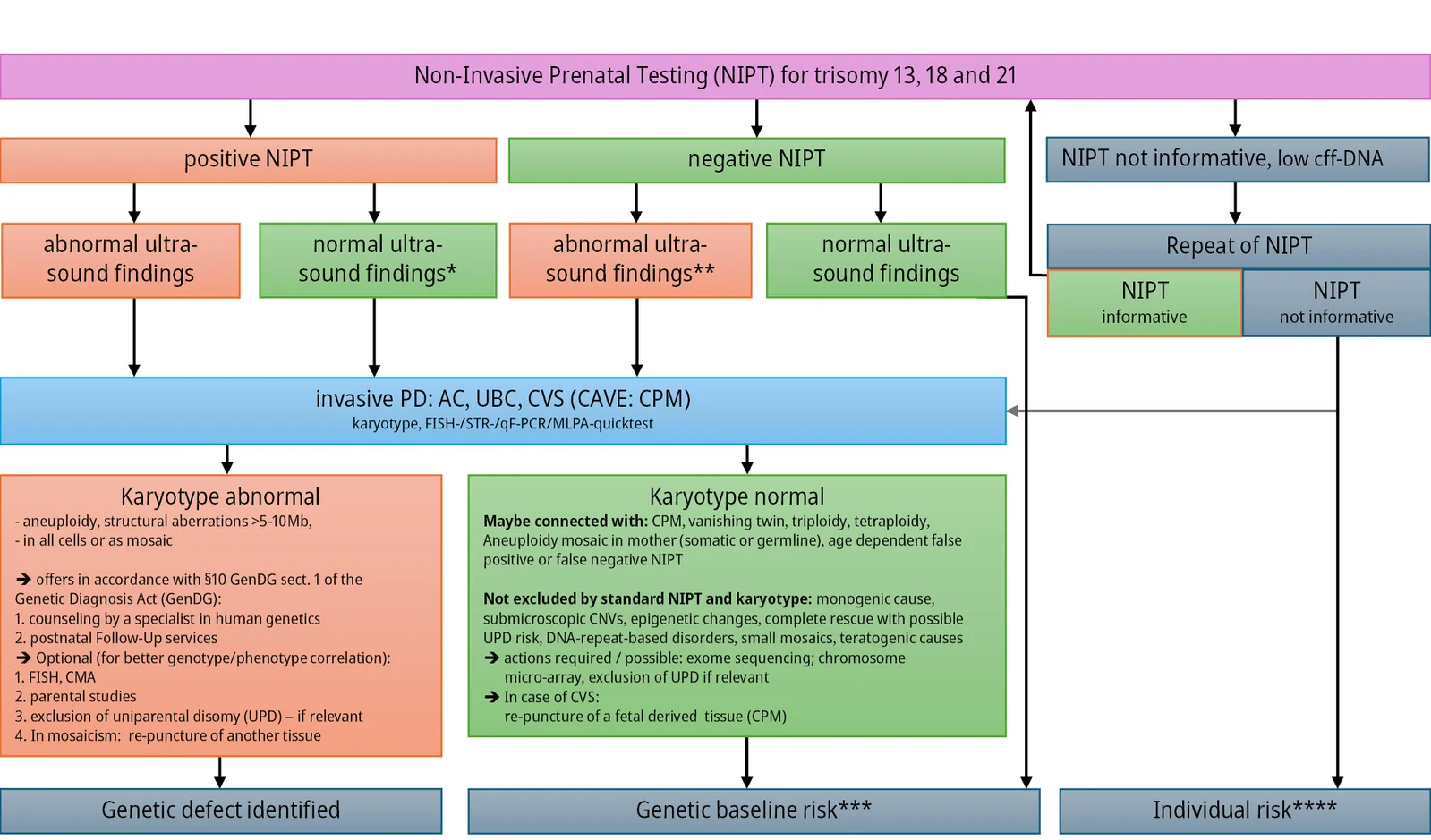
A NIPT for trisomies 13, 18 or 21 may give a positive, a negative or a non-informative result (due to low cff-fraction). In combination with sonography, it may be decided to do an invasive test, which either finds a genetic defect in the foetus, or just can provide a hint on a genetic baseline or an individual risk The asterisks in the scheme are a hint on the following content: * Investigation of a possible mosaic finding; ** In cases of abnormal ultrasound findings, diagnostic amniocentesis should generally be offered rather than NIPT [17; 23]; *** May vary depending on family history (e.g., in cases of consanguinity); **** More common in obese women; may indicate insufficient placental supply and lead to pregnancy complications [24–25], which may be indication for more intense gynaecological monitoring.

### Diverse target regions of different NIPT tests and the reliability of the tests

Many different providers offer NIPT, including tests developed in-house, and the screening can include testing of different target regions. Without claiming to be exhaustive, the authors identified the following variants of ‘standard NIPT screening’ [16]:

i) exclusive test for trisomies 13, 18 and 21;
ii) test for trisomies 13, 18, 21, X and Y;
iii) test for trisomies of all 22 autosomes, (X and Y);
iv) test for trisomies 13, 18, 21, (X and Y), and certain microdeletion syndromes;
v) test for trisomies of all 22 autosomes, (X and Y), as well as certain microdeletion syndromes;
vi) genome-wide analysis for copy number gains or losses;
vii) variants 1–6 with detection of single-gene mutations (see next section of this article), and in case of SNP-based tests also check for tri- or tetraploidy.

In Germany, test variant (i) is reimbursed by health insurance companies since July 2022. Figure 1 schematically depicts the possible outcomes of NIPT screening and the further options for reacting to each test result according to the authors view on diagnostic scenario.

As previously mentioned, the reliability of NIPT depends on the technique used. Furthermore, there is a lack of studies comparing the same samples across different NIPT test platforms. An interesting paper from 2014 is worth mentioning here, in which blood from a non-pregnant woman was sent to five commercial US NIPT providers. Two providers stated a too-low foetal fraction, and three providers issued a normal female result [26].

With regard to NIPT versions that include a test for trisomy 18, it should be noted that screening for aneuploidy of chromosome 18 may also detect a viable form of partial tri- or even tetrasomy of the short arm of chromosome 18. While partial trisomy 18p leads to minimal symptoms, partial tetrasomy 18p leads to a syndrome described in OMIM (https://omim.org/) under the entry #614290.

Other major issues with test results include the fact that NIPT screening reliability varies depending on the stage of the pregnancy [29], the age of the mother [30] and the prevalence of the condition [13, 16]. For trisomy 13, the positive predictive value (PPV) is 0.69, meaning that 44.3 % of women with a positive NIPT result receive false positive information. The values are better for trisomy 21 (PPV: 0.95, percentage: 9.8 %), while they are even worse for trisomy 18 (PPV: 0.84, percentage: 18.5 %), rare autosomal trisomies (PPV: 0.54, percentage: 84.5 %), and sex chromosome aberrations (PPV: 0.64, percentage: 55.5 %) [13]. In 2023, the American College of Medical Genetics and Genomics confirmed these values, along with those of other studies [31 – 32]. Dungan et al. [31] stated that ‘the PPVs for trisomies 18 and 13 are lower than for trisomy 21, largely because of the corresponding lower prevalence of these conditions’, which is a difficult argument to follow. PPVs for microdeletion syndromes are still rarely published. Between 2021 and 2025, four papers provided a real positive rate (falsely given as PPV), which varied between 12.5 % [33], 40 – 50 % [34 – 35], and 88 % [36]. Recently, concerns have been raised that we (the authors of this paper) presented the false positive rate in a potentially misleading manner in previous papers [13–14, 16, 29, 38]. We therefore wish to clarify that there are two distinct ways of reporting this metric. One approach, used by Krawczak et al. [37], calculates the false positive rate among all women undergoing testing, yielding a value of around 0.1 %. Alternatively, the rate can be calculated among women receiving a positive NIPT result, yielding a value of around 50 % or higher [30]. The latter measure seems more informative in a clinical context as it implies that approximately half of women with a positive NIPT result are carrying an unaffected foetus. This has important implications for affected individuals, including increased psychological stress and potentially the need for invasive prenatal diagnostic procedures to confirm the screening result. Furthermore, false positive NIPT results may lead to the termination of pregnancies involving unaffected foetuses, as was reported very recently [39].

## New developments

As mentioned earlier, some commercial NIPT approaches include single-gene testing. In recent years, whole-genome sequencing from cfDNA has also been published [3].

It should be noted that the technical, biological, medical and ethical issues of NIPT discussed by us earlier remain valid for single gene testing [13, 16]. These issues, in addition to the aforementioned technical ones, are as follows: Biological issues: Is the result correctly attributed to the foetus, or is the aberration (i) only present in the placenta, (ii) the result of a second foetus, (iii) indicative of an unrecognised malignancy in the mother, or (iv) indicative of cryptic blood mosaicism? Medical issues: (i) Did the couple understand the significance of NIPT? Did they understand that a negative result does not guarantee that the child will be 100 % healthy, and that a positive result may be a false positive? Ethical issues: (i) How reliable is the informed consent? (ii) What about the right to life of the unborn child? [16, 38, 40].

Kypri et al. [21] summarised that cfDNA analyses for single gene disorders are already offered by several providers. They highlight that ‘the analysis of genetic variations can be deduced based on the absence or presence of foreign alleles in the maternal plasma’; this point is discussed in more detail elsewhere [3]. Kypri et al. [21] also state that ‘the detection of point mutations that derive from the mother is still challenging’ and that ‘parental haplotype is utilized to interpret foetal inheritance pattern’ [21]. They also noted that, by 2022, such single-gene-directed tests had been performed and published for beta-thalassaemia, congenital adrenal hyperplasia, haemophilia, RhD blood typing, sickle cell disease and Wilson’s disease. In their 2023 review, Abedalthagafi et al. [41] state that the application of different variants of genome-wide NIPT approaches has enabled the detection of additional monogenic diseases from cff-DNA, including achondroplasia, Apert syndrome, autosomal recessive polycystic kidney disease, Huntington’s chorea, congenital adrenal hyperplasia, cystic fibrosis, Duchenne muscular dystrophy, haemophilia, methylmalonic acidemia, myotonic dystrophy, spinal muscular atrophy, and thanatophoric dysplasia. According to Abedalthagafi et al. [41], the analysis of cases with confirmatory follow-up revealed no false-positive or false-negative results. If this proves to be the case, and if a recent study [42] yields similar results, foeto-placental discordance would occur less frequently for single-gene disorders than for aneuploidies.

At the annual meeting of the European Society of Human Genetics, our evaluation revealed that 35 abstracts dealt with NIPT. The majority of these reported only on copy number variation assessment by NIPT, while only four had single gene mutation detection by NIPT as their topic. One dealt with future CFTR-gene screening [43], one with achondroplasia mutation screening [44], one with screening for genes related to gestational diabetes mellitus [45], and one with the use of artificial intelligence to detect single-gene disorders [46]. Meanwhile, some NIPT providers only offer screening for selected genes such as *CFTR*, *HBB* or *SMN* [47], while others check for between 84 and 100 monogenic disorders [48–49]. Recently, Chinese authors published an attempt at NIPT screening for ‘202 dominant single-gene disorders using an expanded 155-gene panel’ [50]. However, an Italian paper announced the ‘VERA Revolution’ test, which provides ‘a targeted 1,069-gene panel for comprehensive detection of monogenic disorders and copy number variations’ [51]. Interestingly, this concept includes examinations of both maternal and paternal DNA, as well as cff-DNA.

**Figure 2:**
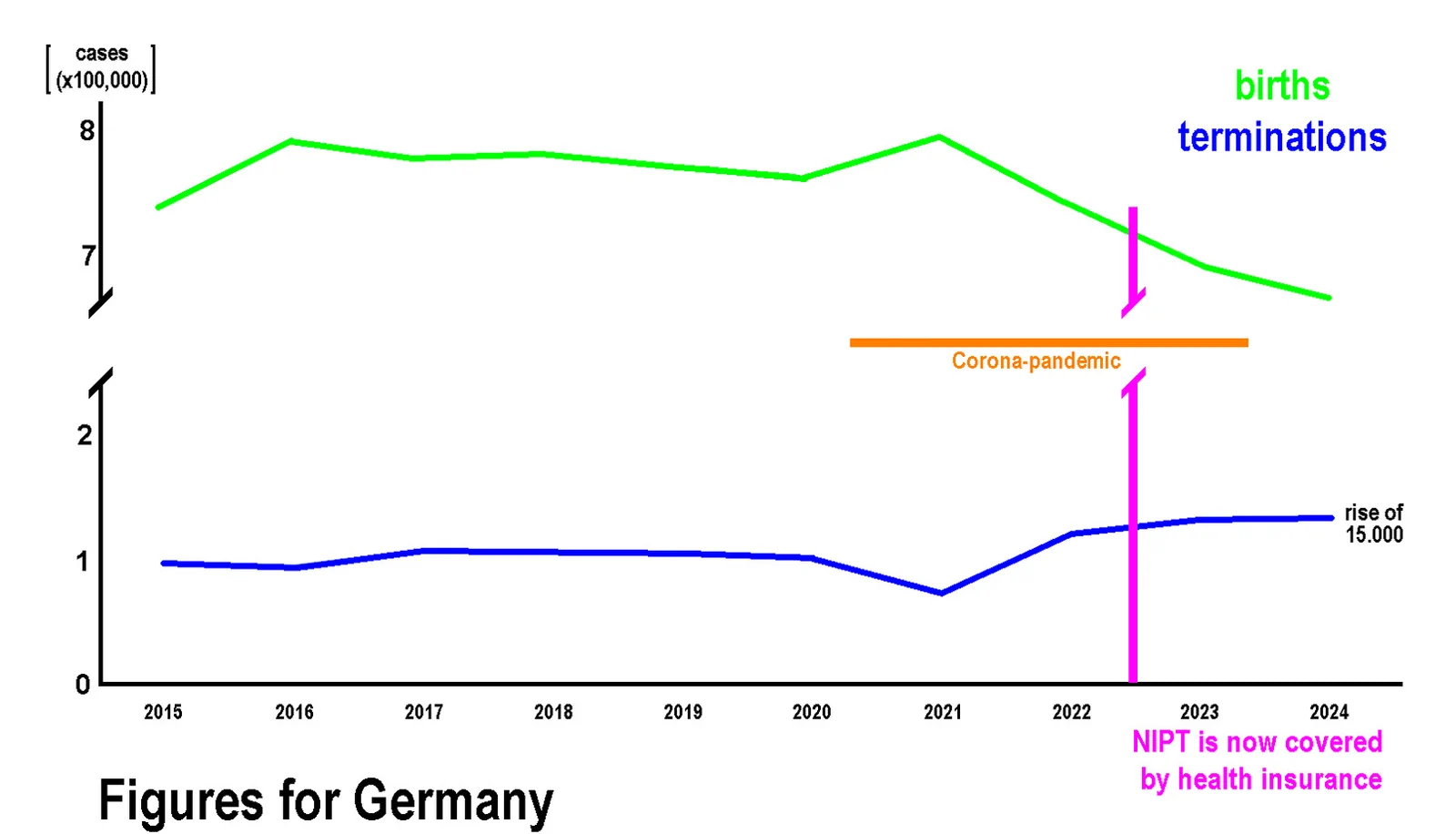
A timeline of births and abortions in Germany is provided. The influence of the Corona pandemic and the coverage of NIPT by health insurance can clearly be seen in this graph. Additionally, the declining birth rate in Germany may be attributed to the political and economic concerns of couples.

## Actual situation in Germany

We previously reviewed NIPT applications and associated peculiarities for Germany in 2022 [38]. The following points need to be added to our previous discussions:

NIPT has been introduced in Germany (and other countries) in the hope of reducing the need for invasive procedures and saving costs. However, according to data published in 2025, neither of these goals seems to have been achieved. NIPT has effectively become a screening test, with 25 % of pregnant women under 26 years old and 75 % of those over 40 taking the test. This accounts for ~25 % of prenatal care costs in Germany, as 50 % of all pregnancies have been screened by NIPT since 2022 [52].

At the same time, the number of invasive PD procedures increased from 0.1 to 2.5 per 1,000 pregnancies, even though they are only reimbursed by German statutory health insurance companies if there is a medical indication [52]. Consequently, the Joint Federal Committee’s (Gemeinsamer Bundesausschuss) goal of using NIPT to avoid invasive prenatal diagnostics [53] has not been achieved.

A very recent study [39] showed that the situation is even more severe: in Germany, only 51 % of positive NIPT tests are followed up with an invasive procedure, and 24 % of pregnancies without an invasive follow-up were terminated. The calculated false positive rate varied with maternal age, ranging from 79 % for women under 30 years old to 34 % for women aged 35 – 40.

While NIPT is performed within the 10^th^ to 12^th^ weeks of gestation, legal abortions are possible (under the counselling requirement) according to § 218 of the German Criminal Code (StGB) without giving any reason up to the 13^th^+6^days^ week mark. Notably, a 9.9 % increase in early abortions was observed in 2022 when NIPT became reimbursable by German statutory health insurance companies. The relative number therefore is even higher as during the same time the birth rates in Germany decreased (Figure 2).

According to § 218 StGB in Germany, termination of a pregnancy is possible without giving any reason at the time of NIPT (10^th^ to 12^th^ week of gestation) under the counselling requirement. Since NIPT was introduced as a paid test in Germany in July 2022, there has been a notable increase in early abortions. This is particularly striking given the dramatic decrease in the general birth rate during this period (Figure 2 – data taken from [54]). Interestingly, 17 % of couples who took the NIPT test cited the main motivation of wanting to learn the gender of the foetus. However, this conflicts with German law. Firstly, sex determination is only a necessary medical requirement in a few cases, and would only be financed by health insurance in these cases. Secondly, according to § 15 GenDG, German gene diagnostic law regulates that solitary sex determination and notification of sex before the 13^th^+6^days^ week is illegal. Violations can be punished with imprisonment or a fine. Therefore, physicians must ensure that they only participate in medically indicated examinations or, at the very least, refrain from communicating the results of gender-specific NIPT before the 14^th^ week of gestation [55].

## Conclusions

Overall, NIPT has been further developed; however, the field still lacks fundamental research into the biological basis of NIPT. The different technical and target-specific variants of NIPT need to be clearly distinguished, and specialist knowledge was already negatively correlated with support for and positivity towards NIPT [56]. Genetic counselling providers need ongoing education that is more scientific than economic in nature, focusing on the advantages and limitations of NIPT. It should be noted that the marketing is highly aggressive, discrediting ‘conventional prenatal tests’ by claiming they provide low accuracy, a 0.5 % risk of abortion and results after 12 weeks of gestation, while presenting NIPT as problem-free. In addition, Săbău et al. [32] state that turnaround times for evaluation of single gene-oriented tests are between a few weeks and one month, a fact not mentioned in advertising. Human genetics is a patient-oriented field rather than a screening-based one. Accordingly, NIPT must be understood as just another gateway for cost-efficient arguments in favour of modern, poorly understood approaches versus established ones [57]. Meanwhile, placenta derived cffDNA is starting to be used for “non-invasive foetal sequencing (NIFS)”, which again – like NIPT – falsely claims to study the foetus, for prenatal screening of almost 23,000 genes [58]. This raises new ethical issues, particularly with regard to the right of the unborn child not to know.

## List of abbreviations

AC: amniocentesis
AFP: alpha-foeto-protein
ß-HCG: ß-human chorionic gonadotropin
cff-DNA: cell free foetal DNA
CPM: confined placental mosaicism
CVS: chorionic villi sampling
NIPT: non-invasive prenatal testing
NT: nuchal translucency
PAPP-A: pregnancy-associated plasma protein-A
PCR: polymerase chain reaction
PD: prenatal diagnostics
PPV: positive predicted value
RhD: foetal rhesus D status
SCA: sex chromosome aberration
SNP: single nucleotide polymorphism
UBC: umbilical cord blood
